# Back-end-of-line-compatible low-voltage operation in Hf_0.5_ Zr_0.5_O_2_ ferroelectric film enabled by *in-situ* lanthanum doping

**DOI:** 10.1093/nsr/nwag049

**Published:** 2026-02-02

**Authors:** Yinchi Liu, Kangli Xu, Shuqi Tang, Handong Zhu, Lin Chen, Shiyou Chen, Wenjun Liu, Peng Zhou

**Affiliations:** College of Integrated Circuits and Micro-Nano Electronics, Fudan University, Shanghai 200433, China; Shaoxin Laboratory, Shaoxing 312000, China; College of Integrated Circuits and Micro-Nano Electronics, Fudan University, Shanghai 200433, China; College of Integrated Circuits and Micro-Nano Electronics, Fudan University, Shanghai 200433, China; College of Integrated Circuits and Micro-Nano Electronics, Fudan University, Shanghai 200433, China; College of Integrated Circuits and Micro-Nano Electronics, Fudan University, Shanghai 200433, China; College of Integrated Circuits and Micro-Nano Electronics, Fudan University, Shanghai 200433, China; College of Integrated Circuits and Micro-Nano Electronics, Fudan University, Shanghai 200433, China; Shaoxin Laboratory, Shaoxing 312000, China; College of Integrated Circuits and Micro-Nano Electronics, Fudan University, Shanghai 200433, China; Shaoxin Laboratory, Shaoxing 312000, China

**Keywords:** lanthanum doping, ferroelectricity, back-end-of-line, low-voltage operation

## Abstract

HfO_2_-based ferroelectric devices have attracted substantial attention in non-volatile memories due to their sub-3 nm scalability and robust ferroelectric properties. However, challenges such as double remanent polarization (2*P*_r_) degradation and high thermal budget have hindered their integration as advanced technology nodes. Here, we developed an *in-situ* lanthanum doping strategy in ferroelectric capacitors to address these issues. By controlling the atomic layer deposition pulses of La_2_O_3_, HfO_2_ and ZrO_2_, the lanthanum-doped HZO (La: HZO) film with different lanthanum concentrations were prepared. An ultra-low thermal budget below 300°C was demonstrated in the 0.44% La: HZO film, while maintaining a pronounced 2*P*_r_ of 27.8 *μ*C/cm^2^. First-principles calculations suggest that lanthanum incorporation promotes the formation of oxygen vacancies, which is believed to stabilize the orthorhombic phase and activate ferroelectricity in the La: HZO films. Furthermore, the back-end-of-line compatible capacitor with La: HZO also exhibits a low operating voltage of 2.0 V, excellent ferroelectricity with 2*P*_r_ of 37.5 *μ*C/cm^2^, rapid switching speed of 446 ns, record-high breakdown voltage of 5.73 V, superior endurance properties over 10^11^ cycles and substantial retention. These results provide new insights for the design of non-volatile memories with low power consumption and high operating speed.

## Teaser

*In-situ* lanthanum doping enables BEOL-compatible HZO ferroelectric capacitors with superior polarization of 37.5 *μ*C/cm^2^ under 2 V operation, rapid switching speed, record-high breakdown voltage and excellent endurance over 10^11^ cycles.

## INTRODUCTION

**Figure 1.**
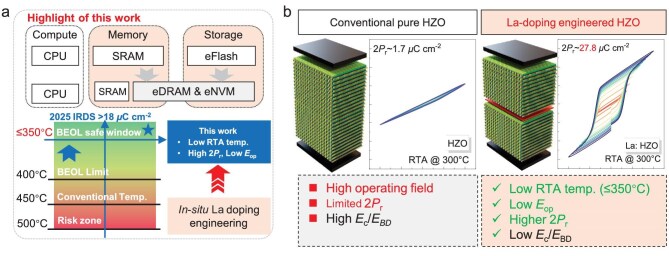
Concept of La-doped HZO (La: HZO) FE capacitors enabling low-voltage operation at BEOL-compatible temperatures. (a) Schematic illustration of the integration challenges in compute/memory/storage systems, highlighting the importance of a BEOL-safe thermal budget (≤350°C) for embedded non-volatile memory. The proposed strategy employs precisely controlled *in-situ* La doping, achieving robust ferroelectricity, high remanent polarization (2*P*_r_), and low operation field (*E*_op_) under low-temperature annealing, as compared to conventional HZO. (b) Conceptual comparison between pure HZO and La-doped HZO devices.

Embedded non-volatile memory (eNVM) has become fundamental to artificial intelligence (AI) hardware by overcoming the limitations of conventional von Neumann architectures through in-memory computing, energy-efficient edge deployment and neuromorphic paradigms [1,2]. To enable these functionalities, advanced compute-memory-storage systems increasingly demand embedded dynamic random-access memory (eDRAM) and eNVM, as schematically illustrated in Fig. 1a. A critical constraint for such integration is the back-end-of-line (BEOL) thermal budget, with a safe processing window of ≤350°C. Satisfying this requirement while achieving strong performance is essential for the next generation of embedded memories.

Recent research has explored various functional materials such as magnetic, ferroelectric (FE), resistive and phase-change materials for next generation NVM [3–6]. Among these, hafnium-based FE materials have attracted particular interest owing to their low power consumption, strong compatibility with complementary metal-oxide-semiconductor (CMOS) processes and proven scalability to below 3 nm [7,8]. Zirconium-doped HfO_2_ (Hf_0.5_Zr_0.5_O_2_, HZO) displays robust ferroelectricity after annealing at ∼400°C, a temperature fully compatible with BEOL thermal budgets, and has become a major focus in NVM research [9–11]. However, when the thermal budget is reduced, conventional HZO films exhibit a reduced FE‐phase fraction, leading to increased operating voltage (*V*_op_), diminished remanent polarization (*P*_r_), slower switching speed and suppressed reliability. These drawbacks limit their large-scale and high-density integration. Although several approaches have been reported to enhance ferroelectricity and reliability at reduced thermal budgets, challenges remain in simultaneously achieving low-voltage operation, strong ferroelectricity, and BEOL compatibility.

To overcome these limitations, a number of strategies have been proposed. Wang *et al*. reported a rhombohedral phase rich in Hf(Zr)_1+x_O_2_ by increasing the Hf:O atomic ratio, achieving an ultra-low coercive electric field (*E*_c_) of 0.65 MV/cm [12]. However, magnetron sputtering faces fundamental scaling limits, highlighting the need for atomic layer deposition (ALD)-based rhombohedral FE films, which have yet to be demonstrated experimentally. Liu *et al*. demonstrated a BEOL-compatible HZO/ZrO_2_/HZO stack that delivers an improved double remanent polarization (2*P*_r_) of 39.6 *μ*C/cm^2^ under 2.0 V and excellent reliability [13,14]. Nevertheless, a comprehensive understanding of switching dynamics remains lacking, and cycling endurance must be improved to satisfy the stability requirements of practical NVM applications. Li *et al*. obtained a low *E*_c_ of 0.6∼1.1 MV/cm and accelerated domain switching [15,16]. Yet, high annealing temperature (>600°C) and low 2*P*_r_ of only 32 *μ*C/cm^2^ require further investigation for BEOL application. Moreover, reducing the thickness of FE films serves as a critical pathway to lower the *V*_op_ and enhance reliability [17–19]. Nonetheless, the concomitant increase in the phase formation energy barrier at reduced thicknesses imposes elevated process thermal budgets, while ferroelectricity degradation presents an additional challenge. In summary, strategies for the co-optimization of ferroelectricity, low-voltage operation, reliability and thermal budget in HZO-based FE memories encompassing process engineering and physical mechanisms still require further exploration.

Here, we demonstrated an *in-situ* lanthanum (La) doping strategy during ALD to induce a controlled concentration of defects and promote the formation of the O-phase under an ultra-low thermal budget of 300°C. Simultaneously, a comprehensive optimization of ferroelectricity and reliability was achieved, providing an innovative and viable solution to the aforementioned challenges, as shown in Fig. 1b. In addition, first-principles calculations, material characterization, and electrical measurements were performed to reveal the mechanism by which lanthanum-doping enhances FE performance and lowers the thermal budget.

## RESULTS AND DISCUSSION

**Figure 2.**
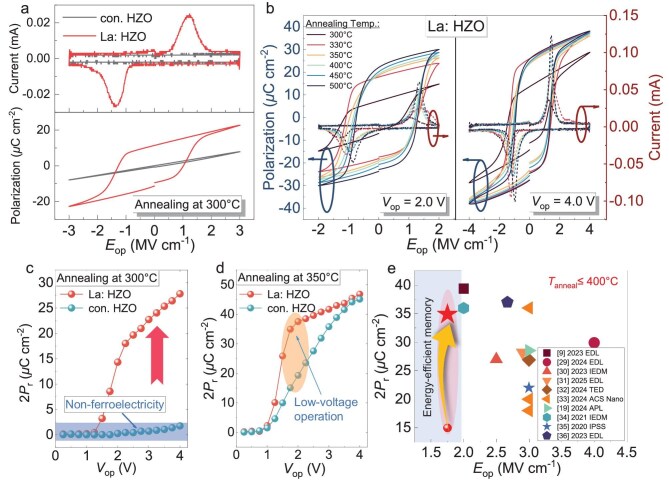
Ultra-low thermal budget and low-voltage operation of La: HZO FE capacitors. (a) Current–electric field (*I*−*E*) and polarization–electric field (*P*–*E*) hysteresis loops for La-doped HZO and conventional HZO capacitors annealed at 300°C. (b) The *P*−*V* and dynamic *I*−*V* curves of FE capacitors based on HZO film with 0.44% La-doping annealed at different temperatures. The 2*P*_r_ of capacitors with La: HZO and conventional HZO annealed at (c) 300°C and (d) 350°C. (e) Benchmark plot of 2*P*_r_ versus operation field (*E*_op_) for this work and previously reported HZO-based FE capacitors annealed at below 400°C, illustrating the superior energy-efficient memory window and industry-leading performance enabled by precise La doping.

The schematic and key process flow of the fabricated capacitor are shown in Fig. S1. By adjusting the ALD cycle ratios of La_2_O_3_, HfO_2_ and ZrO_2_, lanthanum-doped HZO films with lanthanum concentrations ranging from 0.44% to 1.2% were fabricated. Figure S2a and S2b presents the polarization-voltage (*P−V*) and dynamic *I*−*V* curves for lanthanum-doped HZO films with varying lanthanum content under the *V*_op_ of 3.0 V. A significantly reduced coercive voltage (*V*_c_) and enhanced ferroelectricity are obtained in the capacitor with 0.44% lanthanum-doped HZO. As the lanthanum concentration increases, a pronounced splitting of the switching peak is observed in the dynamic *I−V* loops, which is commonly associated with enhanced defect fields and local non-uniformity of defects in HZO films, as shown in Fig. S3 [20–22]. At high La doping level, the defects are expected to favor nonpolar tetragonal/cubic polymorphs and to promote antiferroelectric-like switching features together with a more pronounced wake-up upon cycling [23]. In contrast, an optimized La dose that introduces a moderate defect density could better stabilize the orthorhombic phase and thus enhance ferroelectricity under lower *V*_op_ [24–27]. Figure 2a presents the current–electric field (*I*−*E*) and polarization–electric field (*P*−*E*) loops of FE capacitors with conventional HZO and 0.44% lanthanum-doped HZO (La: HZO) annealed at 300°C. The La: HZO capacitor exhibits a clear FE switching peak and a saturated polarization loop, whereas the capacitor with conventional HZO displays nearly linear dielectric behaviour with negligible hysteresis, indicating the absence of a FE phase at this temperature. Figure 2b presents the *P–E* and dynamic *I−E* curves of FE capacitors with La: HZO annealed at temperatures ranging from 300 to 500°C under the *V*_op_ of 2.0 and 4.0 V, respectively. Noticeable FE hysteresis loops are observed in the La: HZO capacitors at an ultra-low annealing temperature of 300°C. Figure S7 presents the *P*−*V* and dynamic *I*−*V* loops under the *V*_op_ from 1.0 to 4.0 V of the FE capacitors with La: HZO film annealed at different temperatures ranging from 300 to 500°C. Figures S8 and S9 display the *P*−*V* and dynamic *I*−*V* loops for conventional HZO capacitors annealed at 300 and 330°C under the *V*_op_ from 1.0 to 4.0 V. The 2*P*_r_ of the capacitors with La: HZO and conventional HZO annealed at 300 and 350°C are summarized in Fig. 2c and d. With *in-situ* La doping, the La: HZO capacitor delivers a substantial 2*P*_r_ of 27.8 *μ*C/cm^2^ after annealing at 300°C, confirming that ferroelectricity can be realized under an ultra-low thermal budget. A modest increase of the annealing temperature to 350°C further strengthens the FE response and markedly improves low-voltage operation: the 2*P*_r_ increases to 34.9 *μ*C/cm^2^ at 1.75 V and 37.5 *μ*C/cm^2^ at 2.0 V. Therefore, La: HZO capacitors annealed at 350°C are selected for the subsequent breakdown and endurance analyses, as they provide the most favourable trade-off between thermal budget and ferroelectricity. These improvements are attributed to lanthanum doping, which optimizes the crystal structure of HZO film and increases the fraction of the FE phase [13,28]. Figure 2e benchmarks the 2*P*_r_ and *E*_op_ of this work against state-of-the-art HZO-based FE capacitors compatible with BEOL, as reported in recent literature [9,19,29–36]. The La: HZO capacitors fabricated in this study achieve a record-high 2*P*_r_ at the lowest operational field, forming an energy-efficient memory window and highlighting the effectiveness of precise La doping for BEOL-compatible, high-performance FE memory applications.

**Figure 3.**
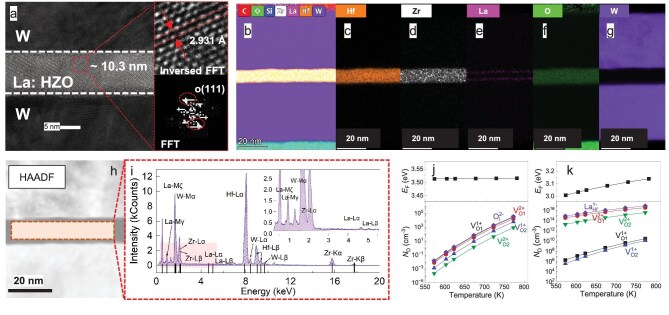
Micro-structure of the capacitor and mechanism for lowing the annealing temperature. (a) Cross-sectional TEM image of the FE capacitor with La: HZO film annealed at 350°C. The insets show the FFT and inversed FFT images of the O-phase. (b–g) EDS elemental distribution maps for Hf, Zr, La, O and W atoms in the capacitor with La: HZO. (h) HAADF image and (i) EDS peak analysis (PA) results. The inset presents a clear energy spectrum of the lanthanum element. Defect concentration and Fermi level in (j) conventional HZO and (k) La: HZO films under different annealing temperatures.

Figure 3a shows the cross-sectional high-resolution transmission electron microscopy (HR-TEM) images of the FE capacitor with La: HZO annealed at 350°C. The insets highlight the (111) plane of the O-phase with a lattice spacing of 2.93 Å in the La: HZO film. A similar (111) plane of the O-phase is observed in HR-TEM images taken from several additional regions across the La: HZO layer as shown in Fig. S4, suggesting that the orthorhombic phase is formed throughout the film thickness. Figure 3b–g presents the energy dispersive X-ray (EDS) elemental distribution maps of Hf, Zr, La, O and W in the FE capacitor with La: HZO annealed at 350°C. The high-angle annular dark field (HAADF) image and EDS peak analysis of the capacitor with La: HZO are shown in Fig. 3h and i. Analysis of the concentration and spatial distribution of lanthanum confirms the successful incorporation of the lanthanum dopant. Consistent with the EDS analysis, X-ray photoelectron spectroscopy (XPS) confirms that adjusting the number of lanthanum ALD cycles effectively tunes the lanthanum content. Figure S5 reports the corresponding cycle counts and the extracted La atomic fractions of 0.44%, 0.82% and 1.2% for the 2-, 4- and 6-cycle La: HZO films, respectively. Figure 3j and k displays the first-principles calculations of defect concentration and Fermi level in conventional HZO and La: HZO films under various annealing temperatures. Lanthanum-doping results in a noticeable increase in defect concentration and a reduction in the Fermi level, indicating an elevated oxygen-vacancy concentration in the La: HZO film which thereby facilitates the stabilization of the O-phase at low annealing temperatures, as shown in Fig. S6.

Ferroelectric polarization reversal consists of two sequential processes, the nucleation of reversed domain and domain growth. The nucleation-limited switching (NLS) model is commonly used to describe switching in polycrystalline materials, as defects such as oxygen vacancies at grain boundaries pin domain walls and impede their motion [37,38]. In such cases, the distribution of switching time follows a Lorentzian function. The Kolmogorov–Avrami–Ishibashi (KAI) model is applicable to single-crystal systems, where domain reversal proceeds by uniform domain wall growth. When the active area of a polycrystalline FE capacitor is reduced to the scale of a single grain, the influence of grain boundary effects become negligible and domain switching becomes rapid and homogeneous [39]. Under these conditions, both models provide equivalent fits to the experimental data.

**Figure 4.**
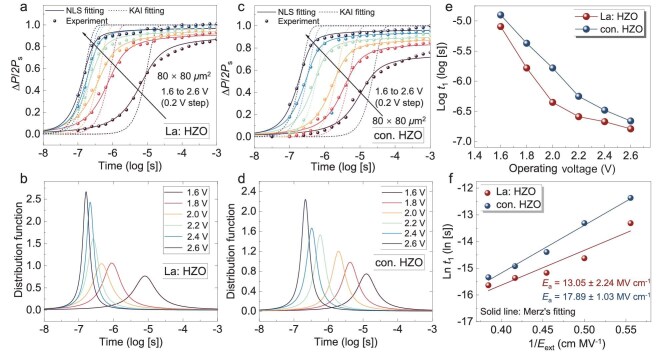
Switching dynamics. Switchable polarization as a function of pulse amplitude and duration time of the capacitors with (a) La: HZO and (c) conventional HZO film. The solid and dashed lines represent the fitting results by the NLS model and KAI model, respectively. (b, d) Lorentzian distribution functions of switching time extracted from (a) and (b). (e) Extracted log *t*_1_ as a function of the operating voltage of the FE capacitor. (f) Calculation of *E*_a_ from the field dependence of the switching time.

Figure 4a and c displays the switching dynamics of capacitors with La: HZO and conventional HZO films, respectively. The solid and dashed lines represent the fitting results by NLS and KAI models. Figure 4b and 4d shows the Lorentzian distribution function of the switching time extracted from Fig. 4a and c. The extraction of the central switching time (log *t*_1_) from Fig. 4b and d as characteristic indexes, and the switching time of the two samples are evaluated and illustrated in Fig. 4e. The polarization-switching time of FE HZO capacitors is jointly determined by the intrinsic FE properties and the applied voltage [40–42]. At a high *V*_op_ of 2.6 V, the FE capacitors with La: HZO and conventional HZO films exhibit a polarization switching time of 162 and 219 ns, respectively. As the *V*_op_ decreases, the switching speed of both capacitors with La: HZO and conventional HZO films slows. Remarkably, the capacitor with La: HZO maintains the switching time of 446 ns and 1.65 *μ*s at 2.0 and 1.8 V, while the capacitor with conventional HZO slows to 1.66 and 4.27 *μ*s under the same conditions. This substantial enhancement in switching speed at low *V*_op_ indicates that domain nucleation is more readily achieved in the La: HZO film. This is attributed to lanthanum-doping, which effectively reduces the domain switching barrier, as depicted in Fig. S10 [43–45]. Furthermore, the activation fields (*E*_a_) were fitted by *Merz’*s empirical formula, as plotted in Fig. 4f. An effective reduction in *E*_a_ from 17.89 to 13.05 MV/cm is realized by lanthanum-doping in the HZO film. This reduction, along with a more uniform domain morphology in the La: HZO film, lowers the external field required for domain mitigation and facilitates dipole reorientation [15].

**Figure 5.**
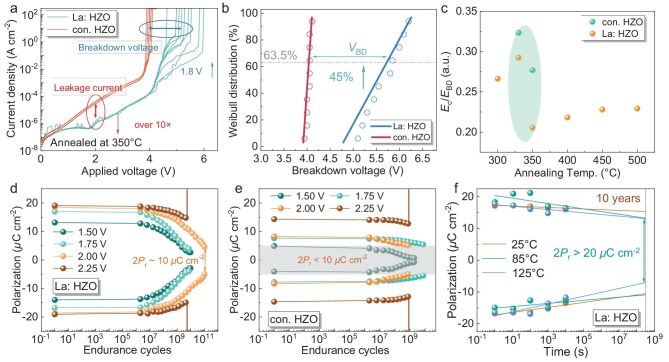
Reliability. (a) TZDB characteristics of capacitors with La: HZO and conventional HZO films. (b) Weibull distribution of breakdown voltages. Ten capacitors were measured in each case to examine their uniformity. (c) The *E*_c_/*E*_BD_ of FE capacitors with La: HZO and conventional HZO films annealed at different temperatures. Endurance characteristics of FE capacitors with (d) La: HZO and (e) conventional HZO films. (f) Retention time of the capacitor with La: HZO under different baking temperatures.

The reliability of the FE capacitors, including time-zero dielectric breakdown (TZDB), endurance properties and retention characteristics was evaluated. Figure 5a and b shows the TZDB characteristics of capacitors with La: HZO and conventional HZO films and the corresponding Weibull distribution of breakdown voltages, respectively. Ten capacitors were measured in each case to assess uniformity. Compared to the capacitor with conventional HZO, the capacitor with La: HZO demonstrates a reduction in leakage current by more than one order of magnitude and a 45% increase in breakdown voltage (*V*_BD_). This improvement is attributed to La-induced oxygen vacancies, which stabilize the ferroelectric orthorhombic phase and tend to be more homogeneously distributed within the O-phase matrix, thereby suppressing percolative leakage paths [46]. The TZDB Weibull distribution of La: HZO capacitors becomes more dispersed, which could be attributed to the higher sensitivity of the breakdown-limiting paths to local variations in La concentration, phase fraction and interface quality in the La: HZO film. The TZDB results of the FE capacitors with La: HZO film annealed at various temperatures from 300 to 500°C are presented in Fig. S11. The La: HZO capacitors annealed at 350°C exhibit the highest characteristic *V*_BD_ of 5.73 V. Figure S12 presents the breakdown voltage and coercive voltage of the FE capacitor with La: HZO film annealed at different temperatures ranging from 300 to 500°C. Figure 5c summarizes the ratio of the *E*_c_ to the breakdown field (*E*_BD_) for capacitors annealed at different temperatures. An exceptionally low *E*_c_/*E*_BD_ of 0.21 is achieved in the capacitor with La: HZO film annealed at 350°C. This leads to an important improvement in the endurance properties of the FE capacitors as shown in Fig. 5d and e. Figure S13 presents the pulse waveform and frequency for the *P–V* and fatigue cycling measurements. Notably, due to enhanced low-voltage ferroelectricity conferred by lanthanum-doping, the La: HZO capacitors maintain a 2*P*_r_ exceeding 10 *μ*C/cm^2^ even after 10^11^ cycles at 2.0 V, whereas the 2*P*_r_ of the conventional HZO falls below 10 *μ*C/cm^2^ after ∼10^9^ cycles. Furthermore, the reduced *E*_c_/*E*_BD_ in La: HZO enables nearly 10^10^ cycles at 2.25 V, while the conventional HZO film undergoes hard breakdown after only ∼10^9^ cycles. Figure 5f illustrates the retention time of the capacitor with La: HZO under different temperatures, with a retention time of over 10 years extrapolated at 125°C.

## CONCLUSION

In summary, we proposed an *in-situ* lanthanum-doping strategy to develop FE capacitors with low annealing temperatures, high ferroelectricity, low operating voltage, fast switching speed and excellent reliability. By varying the lanthanum concentration, a pronounced 2*P*_r_ of 27.8 *μ*C/cm^2^ is achieved under an ultra-low thermal budget of 300°C. Compared to conventional HZO film, the La: HZO film exhibits lower operating voltages of 2.0 V while maintaining an excellent 2*P*_r_ exceeding 37.5 *μ*C/cm^2^ under the annealing temperature of 350°C. First-principles calculations indicate that the introduction of lanthanum promotes oxygen vacancy formation, thereby stabilizing the O-phase and enabling ferroelectricity in La: HZO films. The NLS model was further applied to fit the switching dynamics of both La: HZO and conventional HZO films, revealing an ultrafast low-voltage switching time of 446 ns in the La: HZO film. Moreover, the FE capacitor with La: HZO demonstrates a record-high *V*_BD_ of 5.73 V, superior endurance properties over 10^11^ cycles and excellent retention. These findings provide a feasible method for the synergistic optimization of ferroelectricity and reliability in HfO_2_-based FE devices during the BEOL process, which is highly beneficial for the future integration and application of FE devices.

## METHODS

### Device fabrication

A 50 nm tungsten (W) layer was deposited onto the Si/SiO_2_ substrate using a magnetron sputtering system (PVD 75, Kurt J. Lesker) at 120 W sputter power. Subsequently, the La: HZO and conventional HZO films were grown by PEALD (TFS 200, Beneq) at 250°C, utilizing TEMAHf, TEMAZr, La-FMD and oxygen plasma as Hf, Zr, La and oxygen sources, respectively. By controlling the pulses of HfO_2_, ZrO_2_ and La_2_O_3_, La: HZO films with different lanthanum concentrations were achieved. Afterward, a 50 nm W layer was formed by a magnetron sputtering system, followed by photo-lithography and dry etching yielding an area of 80 × 80 *μ*m^2^. Finally, the FE capacitors were annealed at temperatures ranging from 300 to 500°C in nitrogen atmosphere using rapid thermal annealing.

### Characterization

The switching dynamics and endurance properties were characterized with a TF analyzer 3000. The ferroelectricity and retention characteristics were measured with a Radiant Workstation ferroelectric tester. The time-zero dielectric breakdown was measured by a semiconductor analyzer (Keysight B1500A).

## Supplementary Material

nwag049_Supplemental_File
