# Supplementary material for: Back-end-of-line-compatible low-voltage operation in Hf0.5 Zr0.5O2 ferroelectric film enabled by in-situ lanthanum doping
Source: Natl Sci Rev. 2026 Feb 2;13(6):nwag049. doi: 10.1093/nsr/nwag049 (PMC13045701; doi:10.1093/nsr/nwag049)
Supplement: nwag049_Supplemental_File [file nwag049_supplemental_file.pdf]

Supplementary information

Back-End-of-Line-Compatible Low-Voltage Operation in $Hf_{0.5}Zr_{0.5}O_2$ Ferroelectric Film Enabled by *in-situ* Lanthanum Doping

Yinchi Liu[1, 2†], Kangli Xu[1†], Shuqi Tang[1†], Handong Zhu[1], Lin Chen[1], Shiyou Chen[1], Wenjun Liu[1, 2*], and Peng Zhou[1, 2*]

[1]College of Integrated Circuits and Micro-Nano Electronics, Fudan University, Shanghai 200433, China

[2]Shaoxin Laboratory, Shaoxing 312000, China

†Equally contributed to this work.

*Corresponding author. Email: wjliu@fudan.edu.cn; pengzhou@fudan.edu.cn

Device Fabrication

Figure S1a display the key process flow of HZO-based FE capacitors. By controlling the plasma-enhanced atomic layer deposition (PEALD) cycle ratios of HfO_2, ZrO_2 and La_2O_3, the lanthanum-doped HZO (La: HZO) films with different lanthanum concentrations were prepared[1,2]. The La: HZO and conventional HZO films were deposited using PEALD system at 250°C, with TEMAHf, TEMAZr, La-FMD and oxygen plasma as Hf, Zr, La and oxygen sources, respectively. Figure S1b summarizes the ALD cycle sequences used for the HZO and La: HZO thin films. The HZO-based films were grown by PEALD with a 50W O_2 plasma, using 1 s precursor pulses for both Hf and Zr, followed by 5s N_2 purges and 5s O_2 exposures, resulting in a growth-per-cycle (GPC) of ~1.1Å. The conventional HZO film was deposited using 98 binary cycles of (one HfO_2 cycle + one ZrO_2 cycle), corresponding to a nominal Hf: Zr cycle ratio of 1:1. For La-doped HZO, two identical super-cycles were employed with a nominal La:(Hf+Zr) cycle ratio of 1:48, i.e., one La_2O_3 ALD cycle inserted after every twelve (one HfO_2 + one ZrO_2) sub-cycles.

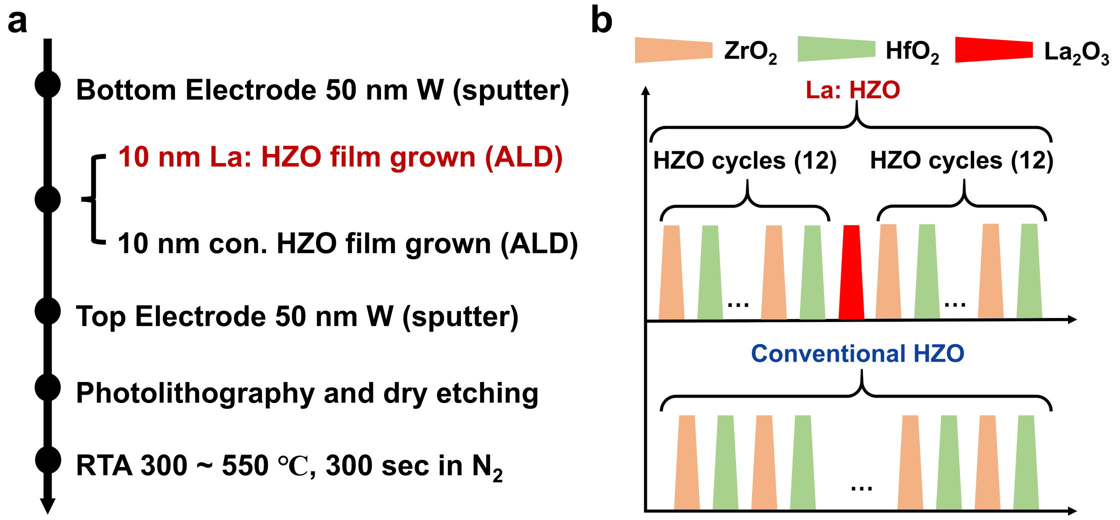

Figure S1. Key process flow of the FE capacitors with La: HZO and conventional HZO films.

First-principles calculation method

All first-principles calculations were performed using density functional theory (DFT) as implemented in the Vienna Ab initio Simulation Package (VASP)[3]. The Heyd–Scuseria–Ernzerhof (HSE) hybrid functional was employed to obtain an accurate band gap and defect energy levels, together with projector augmented-wave (PAW) pseudopotentials[4]. A plane-wave cutoff energy of 520 eV was used throughout the calculations. Defects were modeled using a 96-atom supercell, and Brillouin-zone integration was performed using a single Γ-point[5].

Defect concentrations at different annealing temperatures were first computed under thermodynamic equilibrium by solving the charge-neutrality condition including all defect species. During cooling to the 300K operating temperature, the total concentration of each defect type was fixed, and only the distribution among different charge states was updated. This approach yields the final defect concentrations and Fermi-level positions reported in this study.

The definition of “defect” and “Fermi level”

The equilibrium concentration of each defect state was evaluated by solving the charge-neutrality condition within the HZO layer. For a defect α in charge state q, its concentration is

$$N(a, q) = N_{site} g_q exp\left[\frac{-\Delta E_f}{k_B T}\right]$$

where N_{site} is the number of available lattice sites, $g_q$is the degeneracy factor, and ΔE_f is the Fermi-level–dependent formation energy.

The Fermi level E_f and defect concentration are determined self-consistently by

charge neutrality condition:

$$n_0 + \sum_{a,q<0} [(-q) * N(a,q)] = p_0 + \sum_{a,q>0} [q * N(a,q)]$$

where n_0 and p_0 are the free-electron and free-hole concentrations.

Following common practice for oxide semiconductors, defect concentrations were first computed at the high growth temperature, assuming thermodynamic equilibrium. The total concentration of each defect species was then fixed, and the charge-state distribution was re-evaluated at the device operating temperature based on the updated Fermi level. This procedure yields the final equilibrium defect concentrations and the corresponding Fermi-level position used in Figure 3.

Ferroelectric properties

Figure S2a and S2b show the *P-V* and dynamic *I-V* curves of lanthanum-doped HZO films with varying lanthanum content annealed at 500°C. A significantly reduced coercive voltage (V_c) and enhanced ferroelectricity are obtained in the capacitor with 0.44% lanthanum-doped HZO. As the lanthanum concentration increases, pronounced splitting of the switching peak is observed in dynamic *I–V* loops.

Figure S3 presents the O 1s XPS spectra of the conventional HZO and La: HZO films with increasing La ALD cycles. The La: HZO films used for XPS analysis were prepared following the same flow of capacitors. Specifically, the samples with the W top electrode cap were annealed. After annealing, the W electrode was removed by wet etching, followed by thorough DI-water rinsing and N_2 drying. The O 1s XPS spectra were then acquired on the exposed La: HZO surface.

The spectra can be deconvoluted into a main peak at ~530eV associated with lattice oxygen in Hf–O/Zr–O bonds and a higher binding-energy shoulder attributed to non-lattice oxygen, including defective or weakly bonded oxygen species. With increasing La content, the fitted lattice oxygen fraction decreases from 80.7% in the HZO reference to 70.5%, 61.1% and 53.6% for the La: HZO (1:24), (1:12) and (1:8) samples, respectively, while the higher-binding-energy "non-lattice" component increases correspondingly from 19.3% to 29.5%, 38.9% and 46.4%. Considering the surface sensitivity of XPS and the fact that the O 1s spectra were acquired after W-capped annealing followed by wet removal of the W electrode, this non-lattice component could include contributions from surface -OH/CO_x species introduced during post-etching and ambient exposure. The evolution of the lattice and non-lattice components with La content suggests that La incorporation significantly modifies the local oxygen environment in HZO. This is consistent with previous experimental and theoretical reports that La doping can promote oxygen-vacancy related defects and alter vacancy energetics and charge states in HZO films[6–9].

Figure S4a-S4d show cross-sectional high-resolution transmission electron microscopy (HR-TEM) images of the FE capacitor with La: HZO annealed at 350°C, taken from four representative regions across the film. In each panel, the red dashed box marks a crystalline area in the La: HZO film, and the corresponding inset displays the fast Fourier transform pattern highlighting the (111) planes of the orthorhombic phase with a lattice spacing of ~ 2.93Å. The consistent observation of the O (111) lattice

fringes in all four regions indicates that the orthorhombic phase is uniformly formed throughout the La: HZO film.

Figure S5a compares the La 3d XPS spectra of the conventional HZO film (Sample 1) and La: HZO films grown with increasing numbers of La ALD cycles (Samples 2-4, corresponding to La: HZO ratios of 1:24, 1:12 and 1:8). The La 3d peaks around 835-855eV is absent in the conventional HZO and the intensity are increased as 2, 4, 6 La cycles were incorporated in La: HZO films, in which the extracted La atomic fractions are 0.44%, 0.82%, and 1.2%, respectively. Figure S3b schematically illustrates the ALD super cycle used to prepare the La: HZO (1: 24) stack, in which twelve HfO_2/ZrO_2 cycles are first deposited to build the HZO host layer, followed by one La_2O_3 cycle for La incorporation. This HZO–La_2O_3 sequence is then repeated twice to yield an overall thickness of approximately 10 nm while preserving the targeted La to (Hf + Zr) cycle ratio.

Figure S6a-S6c show the formation energies of both intrinsic defects and lanthanum-related defects varied with the Fermi level (E_F). In conventional HZO films, the E_F is determined by the positively charged oxygen vacancy (V_O^{2+}) and the negatively charged interstitial oxygen (O_i^{2-}). However, in La: HZO films, the charged substitutional defect La_{Hf}^{-}, in which a lanthanum atom substitutes for a hafnium, has a lower formation energy and thus becomes the dominant negatively charged defect, resulting in a decrease in E_F. As the lanthanum concentration increases, the formation energy of the La_{Hf}^{-} defect further decreases, causing E_F to shift toward the valence band.

The shifted E_F promotes the generation of oxygen vacancies in La: HZO films, which is believed to stabilize the formation of ferroelectric O-phase and activate ferroelectricity in the FE films[10,11]. However, over doping with lanthanum may lead to an excessive formation of oxygen vacancies, which in turn induces severe domain pinning[12].

Figure S7 and Figure S8 show the polarization-voltage (*P*-*V*) of capacitors with La: HZO and conventional HZO films, respectively. The coercive electric-field (E_c) of FE capacitors with La: HZO and conventional HZO films annealed at 300, 330 and 350℃ are summarized in Figure S9. All measurements were conducted at room temperature with a frequency of 1kHz. As the annealing temperature is reduced from 500°C to 300°C, the FE capacitor with La: HZO film continue to exhibit pronounced ferroelectric hysteresis, while the FE capacitors with conventional HZO film lose ferroelectricity when the annealing temperature reduced to 300°C. This demonstrates that *in situ* lanthanum doping strategy allows for lower annealing temperatures in FE capacitors and enhances their ferroelectric properties.

Figure S13 illustrates the pulse sequence and frequencies used for device testing: an initial *P*-*V* measurement and the fatigue cycling. For the *P*-*V* measurements, a low-frequency bipolar triangular pulse waveform with a frequency of 1kHz was applied to the capacitor to record the *P*-*V* characteristics before and after cycling. For fatigue cycling, a continuous train of bipolar rectangle pulses with a frequency of 1MHz was used to repeatedly switch the polarization state of the device and accelerate fatigue degradation between the *P*-*V* measurement.

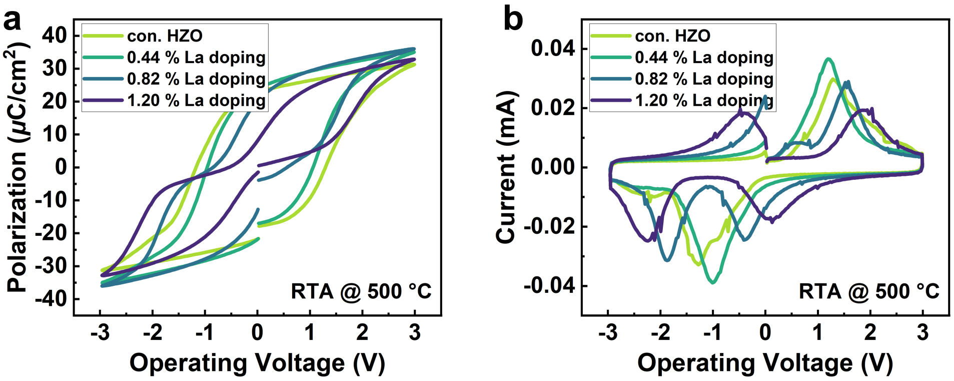

Figure S2. (a) *P-V* and (b) dynamic I-V curves of lanthanum-doped HZO films with varying lanthanum content annealed at 500°C.

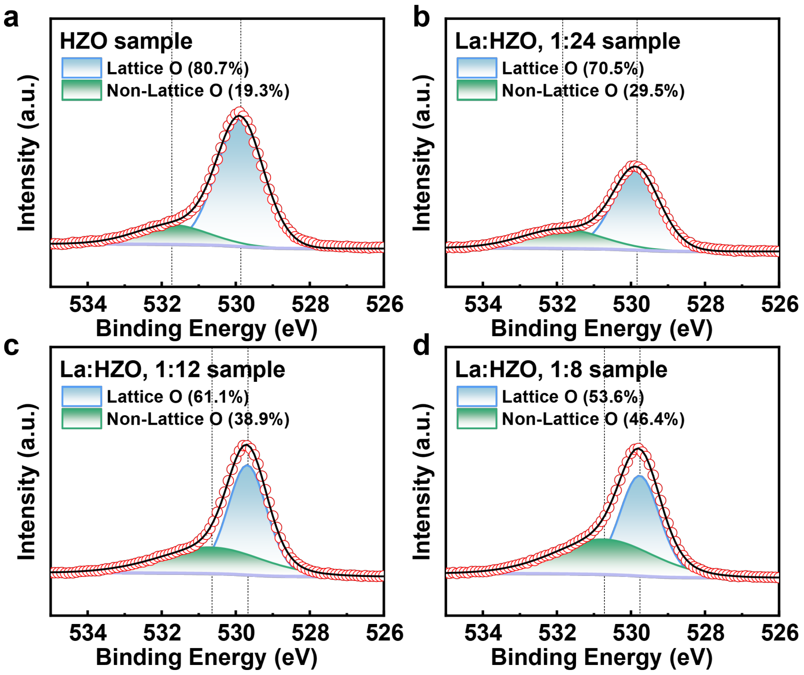

Figure S3. O 1s XPS spectra of (a) the conventional HZO film and La: HZO films with different La ALD cycle ratios: (b) La: HZO = 1:24, (c) La: HZO = 1:12, and (d) La: HZO = 1:8.

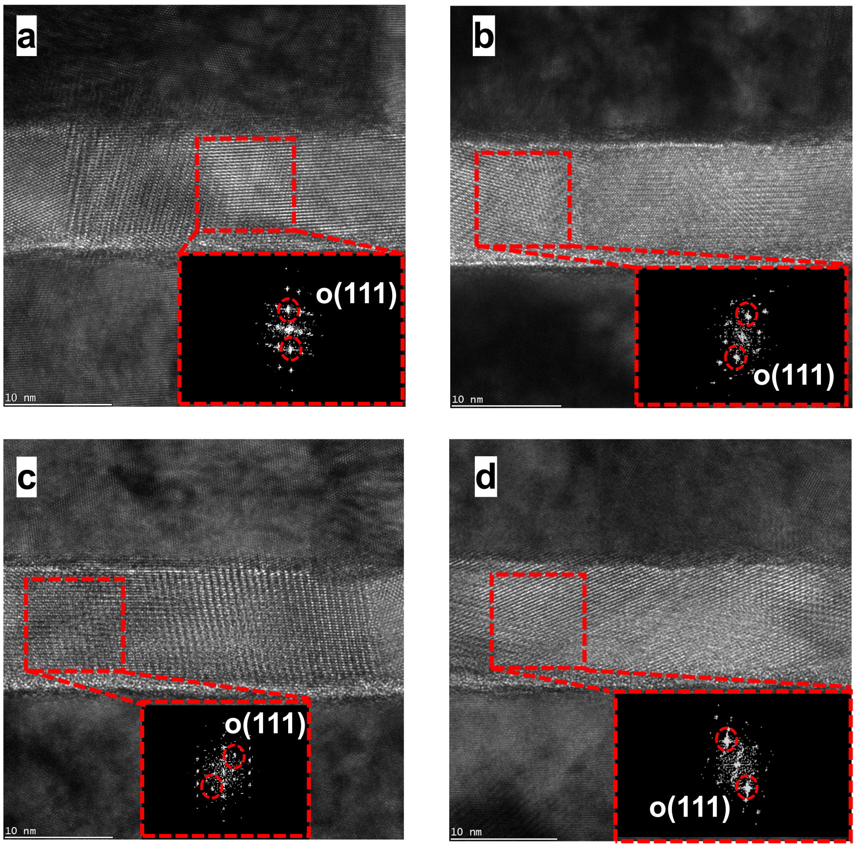

Figure S4. (a)-(d) Cross-sectional TEM image of the FE capacitor with La: HZO film annealed at 350°C. The insets show the FFT and inversed FFT images of the O-phase.

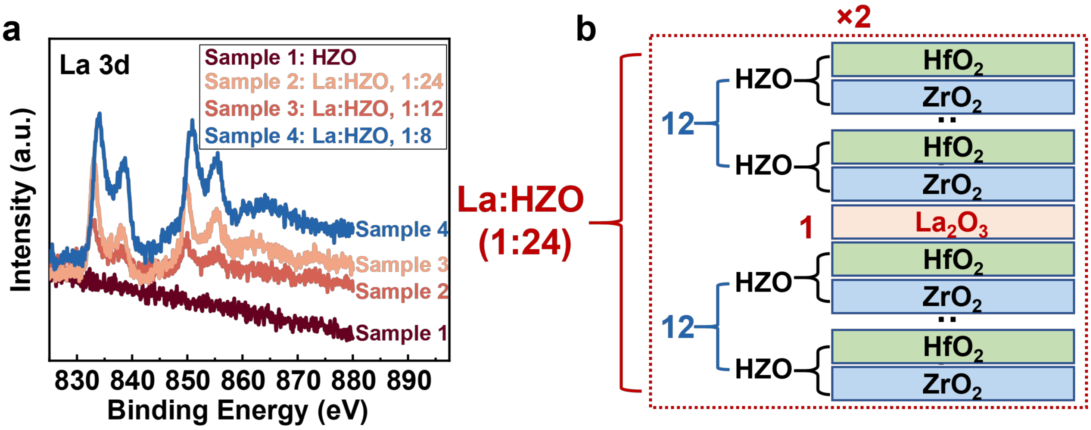

Figure S5. (a) La 3d XPS spectra of the reference HZO film and La-doped HZO films with different La ALD cycles. (b) Schematic diagram of the ALD process.

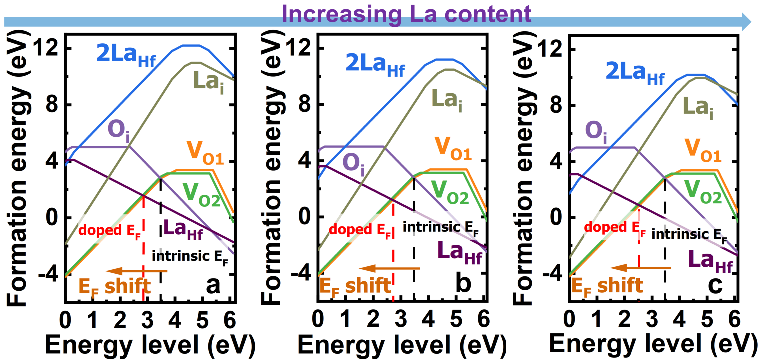

Figure S6. (a)-(c) Defects formation energy in HZO film as a function of lanthanum content. The major defects include intrinsic oxygen-related defects O_i (interstitial oxygen), V_{O1} (threefold-coordination oxygen vacancy), V_{O2} (fourfold-coordination oxygen vacancy), as well as lanthanum-related defects, La_{Hf} (a lanthanum atom substituting for a hafnium), $2La_{Hf}$ (two lanthanum atoms substituting for one hafnium), and La_i (interstitial lanthanum).

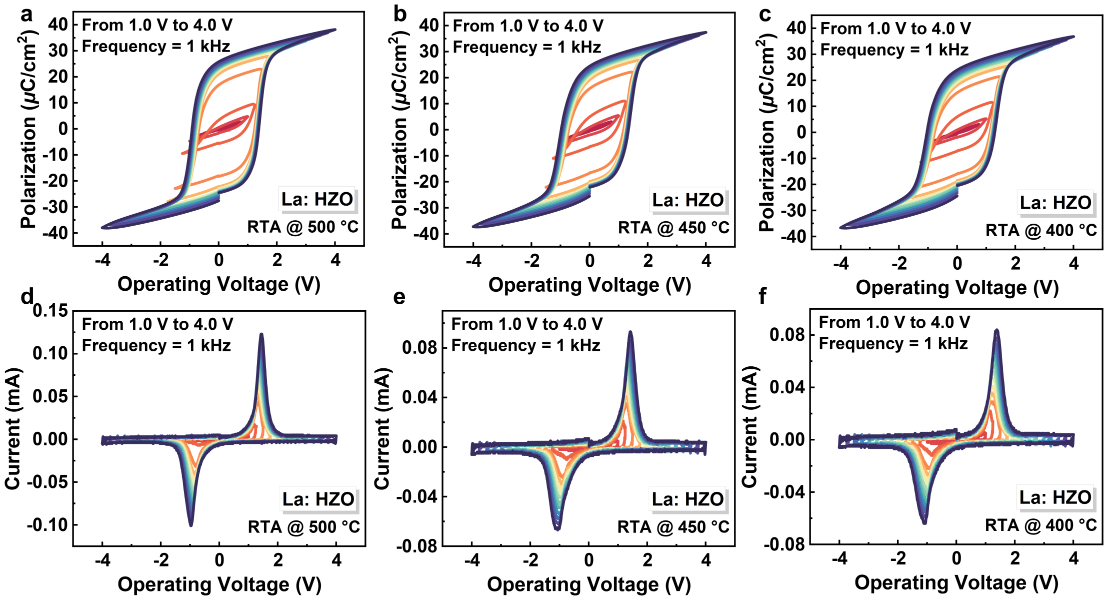

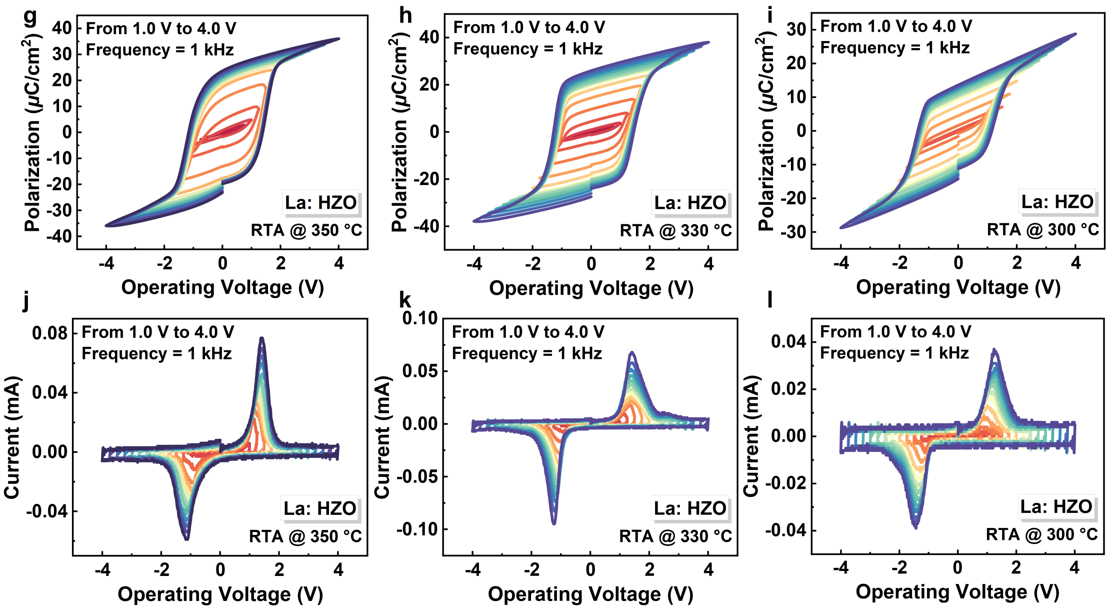

FigureS7. (a)-(l) *P-V* and dynamic *I-V* loops under the V_{op} from 1.0 to 4.0V of the FE capacitors with La: HZO film annealed at different temperatures from 300 to 500℃.

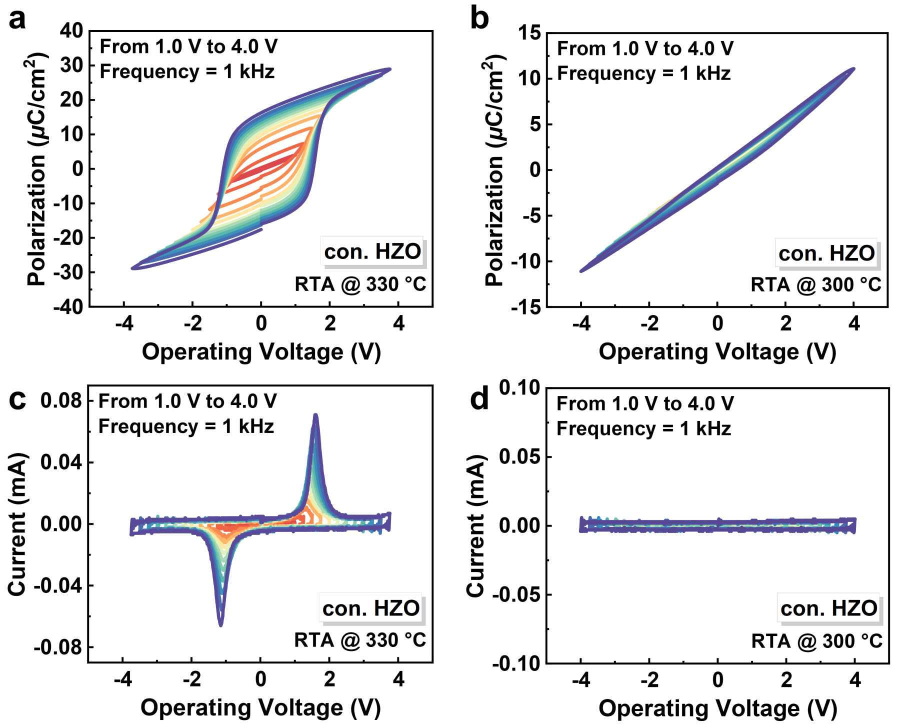

FigureS8. (a)-(b) *P-V* and (c)-(d) dynamic *I-V* loops under the V_{op} from 1.0 to 4.0V of the FE capacitors with conventional HZO film annealed at 300 and 330℃.

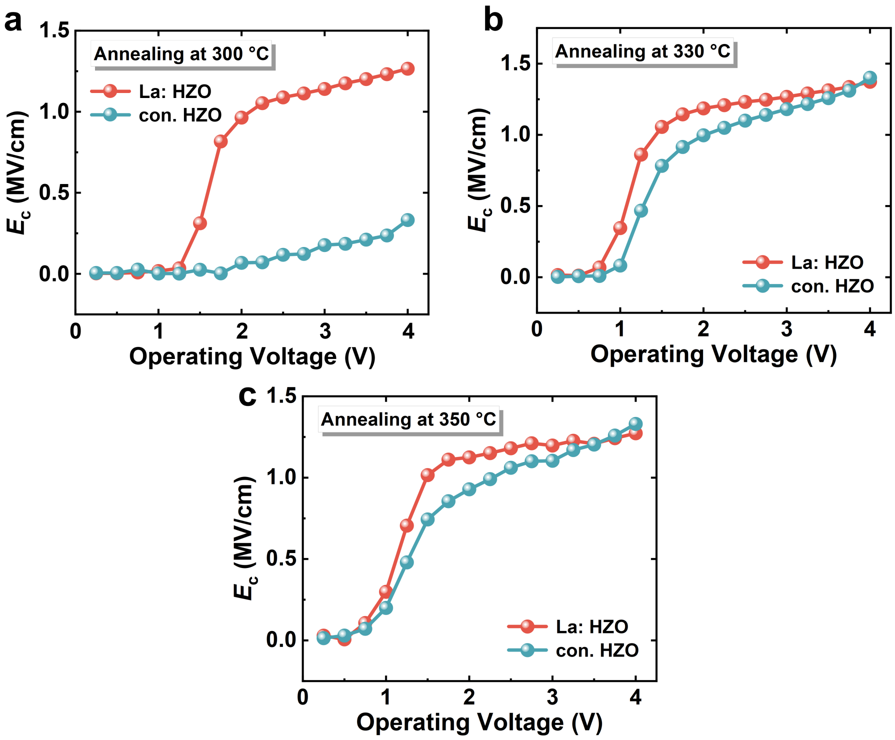

FigureS9. The E_c of the FE capacitors with La: HZO and conventional HZO films annealed at (a) 300, (b) 330 and (c) 350°C.

Switching barrier

All polarization switching speed measurements were conducted at room temperature using electrodes with an area of 80 × 80 μm^2. The fitting procedures for both the nucleation-limited model (NLS) and the Kolmogorov–Avrami–Ishibashi (KAI) model were performed using MATLAB software.[13,14]

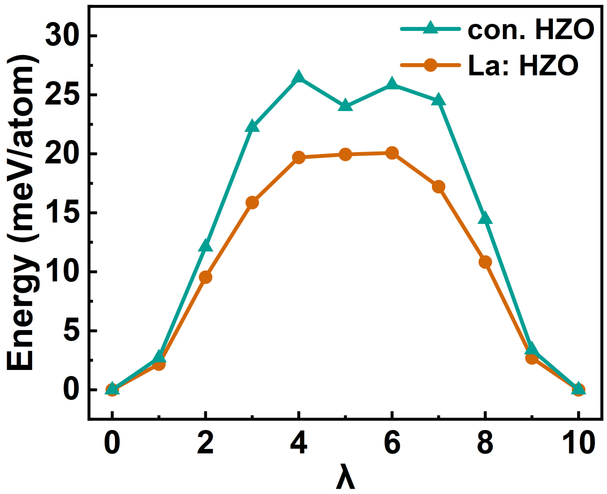

FigureS10. The energy landscape for polar switching of the La: HZO and conventional HZO.

Reliability characteristics

Figure S11a shows the time zero dielectric breakdown (TZDB) results of the FE capacitors with La: HZO film annealed at temperatures ranging from 300 to 500°C. Figure S11b presents the Weibull distribution of breakdown voltages. All TZDB measurements were performed at room temperature. To eliminate initial state variability, a 2.0V polarization-switching pulse was applied to precondition each capacitor before dielectric breakdown characterization. Figure S12 summarizes the breakdown voltage and coercive voltage of capacitors with La: HZO annealed at different temperatures. A record-high breakdown voltage was achieved in the capacitor with La: HZO annealed at 350°C. Table S1 summarizes the film thickness, electrode stack, annealing temperature and breakdown field of representative HZO-based FE capacitors reported in the literature, and compares them with the La: HZO capacitor in this work to allow a fair assessment of the record breakdown performance.

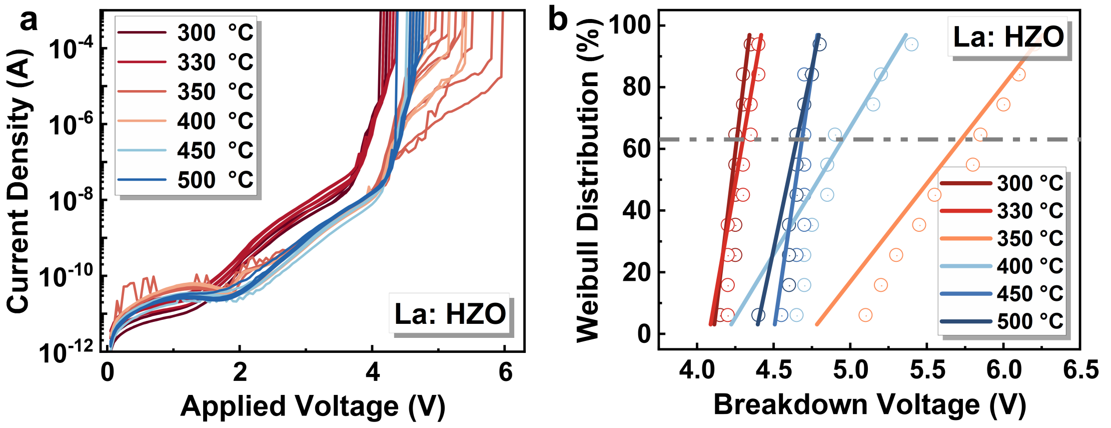

Figure S11. TZDB characteristics. (a) The TZDB results of the FE capacitors with La: HZO film annealed at different temperatures from 300 to 500°C. (b) Weibull distribution of breakdown voltages.

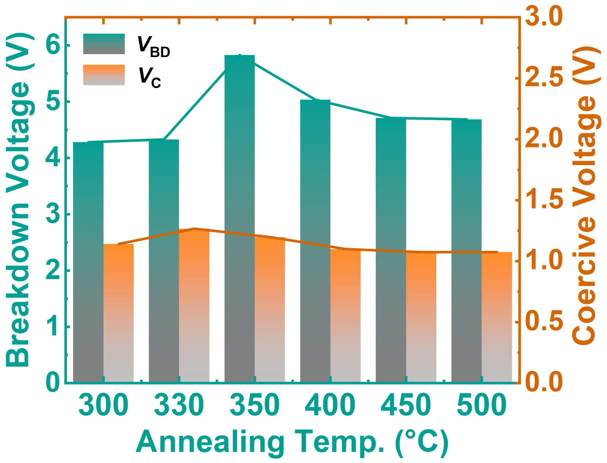

Figure S12. The breakdown voltage and coercive voltage of the capacitors with La: HZO annealed at different temperatures from 300 to 500°C.

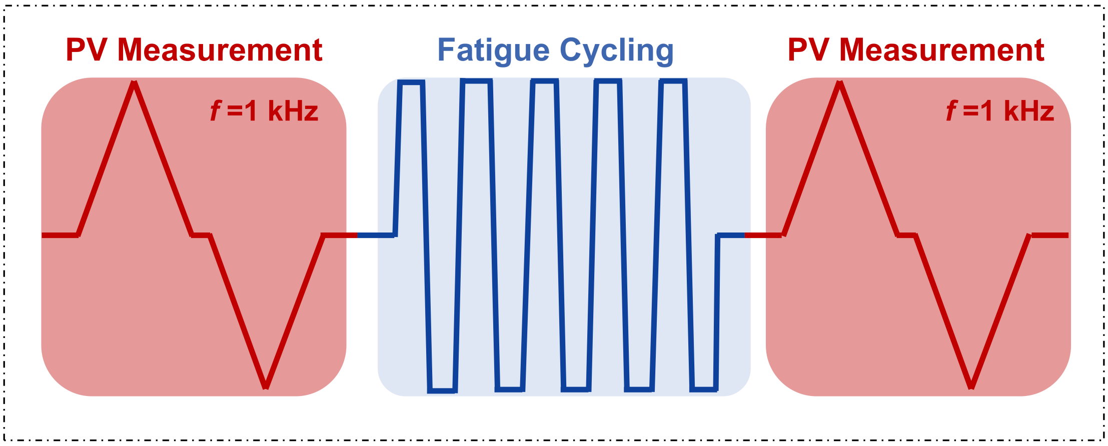

Figure S13. Pulse waveform and frequency for the P–V and fatigue cycling measurements.

Table S1. Literature benchmark of HZO-based ferroelectric capacitors compared with La:HZO device in this work.

Parameter	TED [15]	EDL [16]	TED [17]	EDL [18]	Science [19]	**This work**
Film	HZO/AO/HZO	La: HZO	HZO	HZO/ZO/HZO	r-HZO	**La: HZO**
Film thickness	~10 nm	8 nm	~11 nm	~11 nm	12 nm	**~10 nm**
electrodes	W/W	W/W	TiN/TiN	W/W	TiN/TiN	**W/W**
Process Temp.	600	400 °C	450 °C	350 °C	550 °C	**350 °C**
E_{BD} (MV/cm)	5.25	5	5.1	4.10	4.15	**5.73**
E_c/E_{BD}	0.33	0.42	0.27	0.34	0.16	**0.21**

References

1. Shi W, Zhang D, Zheng Z *et al.* Record-Low Coercive Field in ALD-Grown HZO Ferroelectric Films: Enabling Ultra-low Operation Voltage in World's First 2-Layer 3D Stacked Oxide Semiconductor 1T1C FeRAM. 2024 IEEE International Electron Devices Meeting (IEDM). San Francisco, CA, USA: IEEE, 1–4, December 2024.

2. Xu K, Wang T, Liu Y *et al.* La-Doped HZO (La: HZO) Ferroelectric Devices Toward High-Temperature Application. *IEEE Trans Electron Devices* 2024; **71**: 5375–9.

3. Hafner J, Kresse G. The Vienna AB-Initio Simulation Program VASP: An Efficient and Versatile Tool for Studying the Structural, Dynamic, and Electronic Properties of Materials. In: Gonis A, Meike A, Turchi PEA (eds.). Properties of Complex Inorganic Solids. Boston, MA: Springer US, 69–82, 1997.

4. Heyd J, Scuseria GE. Efficient hybrid density functional calculations in solids: Assessment of the Heyd–Scuseria–Ernzerhof screened Coulomb hybrid functional. *J Chem Phys* 2004; **121**: 1187–92.

5. Wei J, Jiang L, Huang M *et al.* Intrinsic Defect Limit to the Growth of Orthorhombic HfO_2 and (Hf, Zr) O_2 with Strong Ferroelectricity: First-Principles Insights. *Adv Funct Mater* 2021; **31**: 2104913.

6. Chernikova AG, Kozodaev MG, Negrov DV *et al.* Improved Ferroelectric Switching Endurance of La-Doped $Hf_{0.5}Zr_{0.5}O_2$ Thin Films. *ACS Appl Mater Interfaces* 2018; **10**: 2701–8.

7. Perevalov TV, Islamov DR. Impact of lanthanum doping on the electronic structure of oxygen vacancies in hafnium oxide. *Computational Materials Science* 2024; **233**: 112708.

8. Zhou C, Ma L, Feng Y *et al.* Enhanced polarization switching characteristics of HfO_2 ultrathin films via acceptor-donor co-doping. *Nat Commun* 2024; **15**: 2893.

9. Li J-C, Wang F, Hsu H-H et al. Origin of ferroelectricity and anti-ferroelectricity via doping and oxygen vacancy effects in lanthanum-doped hafnium zirconium oxide thin films. *Appl Phys Lett* 2026; **128**: 012903.

10. Zhou Y, Zhang YK, Yang Q et al. The effects of oxygen vacancies on ferroelectric phase transition of HfO_2-based thin film from first-principle. *Computational Materials Science* 2019; **167**: 143–50.

11. Li Z, Tang S, Wang T et al. Effect of Lanthanum-Aluminum Co-Doping on Structure of Hafnium Oxide Ferroelectric Crystals. *Advanced Science* 2025; **12**: 2410765.

12. Li Y-C, Li X-X, Huang Z-Y et al. The Enhanced Polarization Switching Speed and Endurance in $Hf_{0.5}Zr_{0.5}O_2$ Ferroelectric Thin Film by Modulating Oxygen Dose in Ferroelectric Layers. *IEEE Electron Device Lett* 2024; **45**: 829–32.

13. Li Y-C, Huang T, Li X-X et al. Domain Switching Characteristics in Ga-Doped HfO_2 Ferroelectric Thin Films with Low Coercive Field. *Nano Lett* 2024; **24**: 6585–91.

14. Zhou C, Ma L, Feng Y et al. Enhanced polarization switching characteristics of HfO_2 ultrathin films via acceptor-donor co-doping. *Nat Commun* 2024; **15**: 2893.

15. Xu Y, Yang Y, Zhao S et al. Robust Breakdown Reliability and Improved Endurance in $Hf_{0.5}Zr_{0.5}O_2$ Ferroelectric Using Grain Boundary Interruption. *IEEE Transactions on Electron Devices* 2022; **69**: 430–3.

16. Zeng M, Yan S, Liu S *et al.* First Demonstration of High-Temperature Reliability on La: HZO-La:In_2O_3 FeFET with High Endurance of 10^{10} at 125°C. *IEEE Electron Device Lett* 2025; **46**: 1632–35.

17. Zhao Z, Liao Y-T, Chen Y-R et al. C-Axis Oriented HZO on Flat Amorphous TiN Achieving High Uniformity, Breakdown Field, Final $2P_r$, and Endurance. *IEEE Trans Electron Devices* 2025; **72**: 222–7.

18. Liu Y-C, Yang J-N, Li Y-C *et al.* Back-End of Line Compatible $Hf_{0.5}Zr_{0.5}O_2/ZrO_2/Hf_{0.5}Zr_{0.5}O_2$ Stack Achieving $2P_r$ of 39.6 μC/cm^2 and Endurance Exceeding 10^{10} Cycles under Low-Voltage Operation. *IEEE Electron Device Lett* 2024; **45**: 388–91.

19. Wang Y, Tao L, Guzman R *et al.* A stable rhombohedral phase in ferroelectric $Hf(Zr)_{1+x}O_2$ capacitor with ultralow coercive field. *Science* 2023; **381**: 558–63.
